# Advances in Molecular Research of Tracheobronchial Tree Aging: A Systematic Review

**DOI:** 10.3390/ijms26115128

**Published:** 2025-05-27

**Authors:** Constantin Salahoru, Marius Valeriu Hînganu, Paul Salahoru, Delia Hînganu

**Affiliations:** 1 Department of Morpho-Functional Sciences I, Faculty of Medicine, “Grigore T. Popa” University of Medicine and Pharmacy, 700115 Iasi, Romania; salahoru_constantin@d.umfiasi.ro (C.S.); hinganu.delia@umfiasi.ro (D.H.); 2 Department of Surgery I, Faculty of Medicine, “Grigore T. Popa” University of Medicine and Pharmacy, 700115 Iasi, Romania; paul.p.salahoru@umfiasi.ro

**Keywords:** tracheobronchial aging, molecular changes, cellular senescence, mucins (MUC5AC, MUC5B), extracellular matrix (ECM) remodeling, airway smooth muscle (ASM)

## Abstract

Aging affects all tissues in an organism, including the tracheobronchial tree, with structural and functional changes driven by mechanisms such as oxidative stress, cellular senescence, epigenetic modifications, mitochondrial dysfunction, and telomere shortening. Airway aging can be accelerated by intrinsic or extrinsic factors. This review brings together information from the literature on the molecular changes occurring in all layers of the tracheobronchial airway wall. It examines the biomolecular changes associated with aging in the mucosa, submucosa, cartilage, and smooth muscle of the airways. At the mucosal level, aging reduces ciliary function and disrupts mucin homeostasis, impairing mucociliary clearance and contributing to chronic respiratory diseases such as COPD (Chronic Obstructive Pulmonary Disease). Cellular senescence and oxidative stress drive extracellular matrix remodeling and chronic inflammation. Airway cartilage undergoes age-related changes in collagen and fibronectin composition, leading to increased stiffness, while heightened MMP (Matrix Metalloproteinases) activity exacerbates ECM (extracellular matrix) degradation. In airway smooth muscle, aging induces changes in calcium signaling, hypertrophy, and the secretion of pro-inflammatory mediators, further perpetuating airway remodeling. These changes impair respiratory function and increase susceptibility to chronic respiratory conditions in the elderly. By consolidating current knowledge, this review aims to provide a comprehensive overview of the molecular changes occurring in the respiratory tract with aging and to highlight new molecular perspectives for future research on this topic.

## 1. Introduction

Aging is a process that affects all organs and tissues, including the tracheobronchial tree. Because respiratory diseases are the third cause of death globally and most of these are associated with aging, understanding the mechanisms beneath is important for creating a better overall picture. The speed of aging can vary according to certain intrinsic or extrinsic factors. It can differ in different organs and is based on several mechanisms such as epigenetic changes, cellular senescence, oxidative stress, mitochondrial dysfunction, or telomere shortening.

Epigenetic changes studied so far in the context of aging include DNA(deoxyribonucleic acid) methylation, histone modification, chromatin remodeling, noncoding RNA (ribonucleic) regulation, and RNA modification [1]. Among these, DNA methylation is the most studied and is defined as a modification that occurs by the addition of a methyl group at the C-5 position of the cytosine ring by DNA methyltransferases. Methods for measuring DNA methylation have shown accurate results in predicting chronological age as well as biological age, and predictive tools have been developed based on this [2]. Among these, the Horvath clock is one of the most well-known aging-measure tools, having the ability to predict aging systemically in almost all types of human cells and tissues [3]. Using these biological age calculation tools, it has been demonstrated that the acceleration of aging in the tracheobronchial tree can be caused by factors such as the use of electronic cigarettes, tobacco [4], or the presence of the human immunodeficiency virus (HIV) [5]. In chronic obstructive pulmonary disease (COPD), which is known to be a condition associated with an accelerated aging process [6], the epithelial cells of the small airways show aberrant DNA methylation, most of them being hypermethylated, and some of them are hypomethylated [7].

The role of cellular senescence in the respiratory system is a complex one, with negative effects on aging, but at the same time it shows beneficial effects, especially against malignant cellular transformation [8]. The role of cellular senescence in the respiratory system is complex. While it contributes to chronic inflammation and impaired tissue repair, it also plays a protective role against malignant transformation. Senescence is triggered by oncogenic stress and DNA damage, leading to permanent cell cycle arrest through p53 and Rb pathways. This mechanism prevents damaged cells from proliferating and accumulating further mutations, thereby acting as an early barrier to tumor development. In this way, senescence functions as a tumor-suppressor program, particularly relevant in tissues with high exposure to environmental insults, such as the respiratory epithelium [9,10].

It is known that the presence of senescent cells is high in older people and is associated with various chronic respiratory diseases [11]. At the level of airway epithelial cells, cellular senescence has been linked to the initiation of airway remodeling, chronic lung inflammation, and impaired repair processes [12]. These phenomena can lead to the facilitation and progression of pathogens and harmful particles in the lungs [13]. Senescent cells are associated with the senescence-associated secretory phenotype (SASP), which leads to a chronic inflammatory environment in the airways increasing the risk of exacerbations of chronic respiratory diseases such as COPD [14]. *Integrin beta4* (*ITGB4*) deficiency leads to senescence in airway epithelial cells by activating the p53 pathway, both in vivo and in vitro, with implications for airway ciliated cell function [15]. In addition, cellular senescence leads to the remodeling of the extracellular matrix (ECM) and also of the histologic architecture in the respiratory tract [16]. In another study, after airway exposure to fine particles, SASP was demonstrated to influence respiratory smooth muscle (ASM) by inhibiting this phenotype [17]. Cellular senescence is usually associated with other features of aging such as telomere shortening, mitochondrial dysfunction, and defective autophagy [18].

Cellular senescence is usually associated with other features of aging, such as telomere shortening and defective autophagy. Telomeres are repetitive nucleotide sequences at the ends of chromosomes that protect them from degradation. With each cell division, telomeres shorten until a critical length is reached, triggering a DNA damage response that activates the p53 pathway and leads to permanent cell cycle arrest (senescence). In parallel, autophagy—a key cellular degradation pathway—becomes progressively impaired with age, resulting in the accumulation of damaged organelles and misfolded proteins. This defective autophagic flux contributes to mitochondrial dysfunction, oxidative stress, and the SASP, all of which are known to affect airway structure and function [19,20].

Reactive oxygen species (ROS) are oxygen free radicals, which appear as a result of aerobic cellular metabolism [21] and which trigger a cascade of events that perpetuate inflammation, cell damage, and tissue remodeling [22]. Increased ROS can lead to mitochondrial dysfunction [23]. Mitochondrial function at the level of secretory cells and basal cells in the respiratory epithelium is essential for the proper functioning of the mucociliary apparatus, and for example, in COPD, mitochondria show abnormal morphology represented by loss of cristae, branching, fragmentation, and increased mass [24]. Oxidative stress increases with age [25], and the accumulation of ROS at the level of cells and tissues in the respiratory tract leads to their damage with an impact on their function [26].

Together, these mechanisms can contribute to the aging of the tracheobronchial tree with a local and also a systemic impact.

This study aims to summarize the biomolecular changes that take place at the level of the tracheobronchial tree with aging, on each individual layer. Most of the previous studies focus on only one layer of the tracheobronchial wall and the changes brought together with aging at that level, or on the pathological conditions that accelerate these phenomena. We believe the main novelty of this manuscript lies in bringing all this information together to create a unified perspective on tracheobronchial aging phenomena and to guide future research directions.

## 2. Materials and Methods

A systematic search was conducted across multiple electronic databases (PubMed, Web of Science, SciELO, SpringerLink, ScienceDirect, ResearchGate, Wiley Online Library, Lippincott Williams & Wilkins, MDPI, and Bond University Research Portal) for articles published between 2020 and 2025. The Boolean strategy combined the keywords (“aging” OR “senescence”) AND (“tracheobronchial tree” OR “airways”), further refined with (“oxidative stress” OR “epigenetic changes” OR “cellular senescence”) AND (“mucus” OR “mucin” OR “smooth muscle” OR “extracellular matrix” OR “cartilage”). MeSH terms were not used to allow inclusion of newer, non-indexed articles.

**Figure 1:**
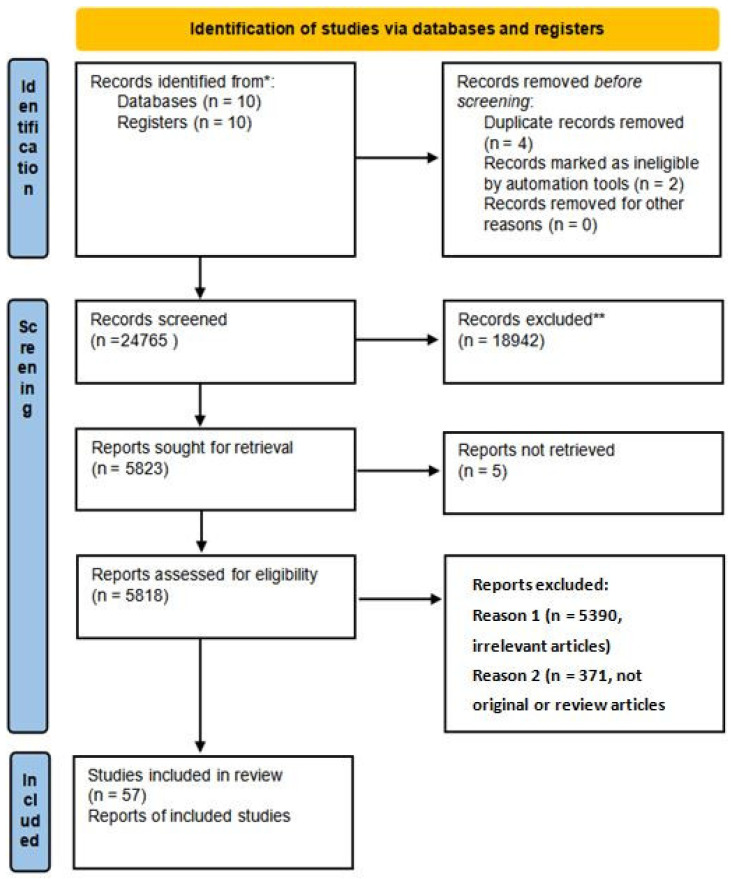
PRISMA 2020 flow diagram, including only database and register searches. (https://www.prisma-statement.org/) * Records identified from databases mentioned in methods. ** Records excluded according to selection criteria from the methods.

The screening involved title and abstract review, selection of English full-texts, and removal of duplicates via automated and manual verification. Eligible studies reported biomolecular aging-related changes in tracheobronchial structures (mucosa, submucosa, cartilage, and smooth muscle). Both original and review articles were considered. Articles with overlapping data, unclear methodology, or ambiguous outcomes were excluded (Figure 1).

Data extraction focused on mechanisms such as epigenetic alterations, senescence, oxidative stress, mitochondrial dysfunction, and telomere shortening, using standardized templates to ensure methodological consistency and reduce bias. The methodology is detailed in the table below and illustrates the number of studies identified, screened, excluded, and included at each stage of the review process (Table 1).

## 3. Results and Discussion

Murine studies were included in this analysis due to their well-established role in aging research and respiratory biology. Mice serve as widely used experimental models because they share key molecular and cellular mechanisms of aging with humans, including oxidative stress responses, epigenetic modifications, cellular senescence, and extracellular matrix remodeling. These models allow for controlled investigations of aging-related processes in a way that would be impractical or unethical in human subjects.

While there are inherent species differences in airway anatomy and immune responses, murine studies provide critical mechanistic insights that complement human data. The inclusion of these studies was carefully considered, and findings from murine models were interpreted in the context of human relevance. Where possible, correlations between murine and human data were made to ensure translational value.

### 3.1. The Mucous and Submucous Layers

The respiratory epithelium represents the first line of defense against inhaled pathogens. This defense is possible through a physical barrier, which involves the proper functioning of the mucociliary apparatus [27]. The tracheobronchial tree contains a pseudostratified ciliated epithelium on the surface of the luminal mucosa, and it is mainly composed of bronchial and bronchiole epithelial cells, including ciliated cells, goblet cells, secretory cells, and basal progenitor cells [16]. The trachea and bronchi are lined with ciliated epithelial cells, and the ciliated cells are covered by a thin layer of periciliary solution that facilitates the movement of the cilia [28]. The normal mucociliary clearance (MCC) time ranges from 7 to 15 min [29,30].

With aging, oxidative stress increases [31], which can lead to the activation of protein kinase Cε (PKCε), and its activation has been associated with the slowing of cilia movement in the tracheobronchial epithelium [32]. Besides these factors, aging is associated with a gradual decrease in the number of cilia and ciliated cells in the respiratory tract [33]. Certain extrinsic factors, such as exposure to cigarette smoke or flavors in e-cigarettes, can accelerate aging by affecting cilia length, with smokers having approximately 13% to 15% shorter cilia than nonsmokers in humans [34] and in mice [35], and exposure to flavoring substances from e-cigarettes leads to a decrease in the number of ciliated cells [36].

An immunohistochemical analysis of the airways of mice demonstrated the presence of age-related gland-like structures (ARGLS) [33,37,38]. However, the presence of these ARGLS was observed only in older mice, suggesting their de novo appearance during life [33].

*SOX2+* (sex-determining region Y-box 2) cells are basal progenitor cells essential for airway epithelial regeneration, particularly in response to injury [39]. *SOX2+* cells are important in maintaining the homeostasis and regenerative potential of the respiratory epithelium [40]. These cells show a decrease in their number with aging and, consequently, a decrease in the regeneration capacity of the respiratory epithelium [41]. In contrast, *p63+* and keratin *5-expressing cells* (*KRT5+*) cells, recognized as distal airway stem cells (DASCs) important in airway epithelial regeneration [42], appear to maintain their numbers and properties with aging in humans, but not in mice [33,41]. This distinction suggests that the decline of *SOX2+* cells is balanced by DASCs *p63+* and *KRT5+* cells, which continue to provide a regenerative reservoir.

Zuo et al. showed that cells from the distal airways expressing DASC *p63/Krt5* are absolutely necessary for lung tissue regeneration after acute lung injuries, and their absence leads to a replacement of lung tissue with fibrous tissue [42].

In a study by Balázs et al. [43], the nasal epithelium was analyzed in children and in older people. The results showed that, at the level of the nasal epithelium, there were no differences between the number of *KRT5+* basal cells between the young and the elderly [43]. A difference in the number of *KRT5+* cells was observed only in pathological aging of the small airways [41], but there are no studies evaluating the number of *KRT5+* cells in the large airways with aging.

With aging, modifications in mucus hydration and rheological properties within the airway may occur. Mucus with abnormal biophysical characteristics is frequently observed in muco-obstructive respiratory diseases such as bronchial asthma, COPD, and cystic fibrosis (CF). These abnormalities arise from alterations in mucin polymer assembly, concentration, macromolecular structure, airway surface hydration, pH, and ion composition [44]. The gel layer of mucus primarily consists of mucin glycoproteins [28]. These mucins are secreted by various types of airway epithelial cells, including basal cells, goblet cells, and ciliated cells [45]. The most important mucins produced in the respiratory tract are classified into two categories: the secreted polymeric mucins, *MUC5AC* and *MUC5B*, and the cell-bound mucins, *MUC1*, *MUC4*, *MUC16*, and *MUC20* [44]. Among the known mucins present in the airway, *MUC5AC* and *MUC5B* are the primary gel-forming mucin glycoprotein [46].

Okuda et al. demonstrated that *MUC5B* is expressed in both the superficial epithelium of the airways and the submucosal glands, with a notable presence in the smaller airways, whereas *MUC5AC* production is restricted to the superficial epithelium of larger, cartilaginous airways [47]. Additionally, it was observed that both *MUC5B* and *MUC5AC* were co-localized with club cell secretory protein 1 (CCSP1)-producing cells in the proximal superficial epithelium, suggesting a shared distribution pattern [47]. The expression of *MUC5B* and *MUC5AC* mucins is elevated in chronic respiratory tract diseases, whereas outside of such conditions, *MUC5B* is the predominantly expressed mucin [48,49].

*MUC5AC* hypersecretion in the airways is a hallmark of pulmonary inflammatory diseases [50]. It has been suggested that *MUC5AC* plays an essential role in the acute-phase airway defense mechanisms [51]. *MUC5AC* has also been associated with inflammation, as demonstrated by studies showing that *MUC5AC*-deficient mice exhibited reduced airway inflammation, while the administration of exogenous *MUC5AC* glycoprotein enhanced inflammatory responses [52,53]. On the other hand, *MUC5AC* enhances mucus viscoelasticity, promoting hydrodynamic coupling between the mucus and periciliary layers, which is essential for effective mucus clearance [54].

*MUC5B* is a crucial gel-forming mucin in the tracheobronchial tree, playing a significant role in mucociliary clearance (MCC) and lung defense [55,56]. It has also been shown that *MUC5B* levels are decreased in aged mice compared to young mice, which has implications for mucociliary clearance (MCC) [57]. In COPD, in murine models, it was demonstrated that MUC5B is the predominant mucin, suggesting an important role in this pathology [57].

*Lactoferrin* (*LTF*) is a multifunctional glycoprotein that belongs to the transferrin family and is capable of binding and transporting iron [58]. It is present in various biological fluids, including breast milk, saliva, tears, mucus, and respiratory secretions [59]. *LTF* possesses immunomodulatory and antioxidant properties, playing a key role in reducing inflammation and oxidative stress in the airways [60]. As an iron-binding protein, *LTF* contributes to the regulation of iron homeostasis by limiting the conversion of free radicals into highly reactive pro-inflammatory species, such as the hydroxyl radical [61]. A study on the effects of *LTF* on cerebral senescence and cognitive function in aged mice demonstrated its ability to reduce oxidative stress and chronic inflammation, leading to an improvement in cognitive function [62]. Studies have shown that *LTF*, in its iron-free form, reduces allergen-induced inflammation, inflammatory cell accumulation, and excessive mucin production in the airways [63]. However, the effects of aging on *LTF* secretion remain unknown, highlighting an important area for future research.

### 3.2. The Cartilaginous Layer

Of the total collagen in the airways, which consists of 28 subtypes, over 80% is represented by subtypes I, II, and III [64]. Type I and type III collagen have a structural role, and their ratio determines the resistance to breaking during stretching. In contrast, type II collagen represents approximately 95% of the collagen found in the cartilage of the trachea and bronchial tubes, playing a key role in facilitating the synthesis of the extracellular matrix (ECM) by chondrocytes [65].

Despite its importance, there are limited studies on how aging affects the ECM of the lungs and tracheobronchial tree. A remodeling of the ECM in the airways of aged mice has been observed, and this remodeling was attributed to a marked increase in the levels of collagen types IV and XVI, as well as a decrease in the level of collagen type XIV [66].

In another study by Ulldemolins et al., the effects of aging on the ECM of the lungs in mice were investigated. The results revealed that fibronectin and type I collagen were significantly increased in older mice compared to young ones, while laminin was reduced in older mice in comparison to young ones [65]. These changes lead to a stiffening of the pulmonary parenchyma and could also be relevant for the tracheobronchial tree.

In conditions such as COPD, which is closely associated with accelerated lung aging, similar alterations in the ECM may contribute to the disease’s progression during aging [6,67,68]. Chronic inflammation and bronchoconstriction lead to significant remodeling of the airway ECM. This remodeling is characterized by excessive deposition of collagen and fibronectin, resulting in the narrowing of the airways and increased resistance to airflow [69].

Matrix metalloproteinases (MMPs) are enzymes that degrade ECM components, such as collagen and elastin. For instance, in the skin, MMP overexpression leads to accelerated aging by breaking down the structural components of the ECM, resulting in loss of elasticity and the formation of wrinkles [70]. In COPD, a condition associated with accelerated aging, plasma and serum levels of *MMP-1* have been shown to increase in proportion to disease severity. This suggests that *MMP-1* plays a role in the progressive remodeling of the airway ECM, contributing to the worsening of the disease [71,72]. Perhaps the most studied MMP in connection with COPD is *MMP-9*. Studies have shown that the levels of this proteinase are increased in patients with COPD, contributing to the degradation of the ECM and promoting inflammation and tissue remodeling in the airways [73,74,75]. MMP-9 has even been proposed as a biomarker for exacerbation risk in patients with COPD, as its elevated levels correlate with increased inflammation and the potential for disease flare-ups [76].

### 3.3. Airway Smooth Muscle

It is known that airway smooth muscle (ASM) plays a crucial role in maintaining branchiomotor tone, and this mechanism is mediated by bronchoconstrictors such as acetylcholine (ACh) and histamine. These molecules increase intracellular calcium concentration, which in turn enhances contractility and leads to bronchoconstriction [77,78,79]. Ca^2^⁺ signaling has been shown to increase with age in several types of smooth muscle cells, contributing to altered muscle contractility and potentially leading to dysfunction in various tissues, including the airways [79,80,81] and ASM [79], showing possible significance in age-related airway changes.

Another role of airway smooth muscle (ASM) is to modulate inflammation and remodeling through the expression and secretion of cytokines and pro-inflammatory mediators. This process contributes to the chronic inflammation and structural changes seen in diseases like asthma and COPD [82]. Senescent airway smooth muscle (ASM) cells are associated with the senescence-associated secretory phenotype (SASP), which leads to chronic low-grade inflammation and ECM remodeling. This process contributes to the persistent inflammation and tissue changes observed in respiratory diseases such as asthma and COPD [83].

ASM cells also express integrins and adhesion molecules such as *intracellular adhesion molecule 1* (*ICAM-1*) and *vascular endothelial cell adhesion molecule 1* (*VCAM-1*). These molecules are upregulated in response to inflammatory cytokines and play a crucial role in facilitating the migration and adhesion of inflammatory cells, contributing to airway inflammation and remodeling [84].

The development of airway smooth muscle hypertrophy is influenced by both mechanical and inflammatory mechanisms. Mechanical factors, such as airway stretching and increased airway resistance, along with inflammatory stimuli, such as cytokines and growth factors, contribute to the thickening and remodeling of the smooth muscle in conditions like asthma and COPD [85]. Mechanical stretch induces hypertrophy through various signaling pathways, including *Wnt*, *GSK3*, *Akt*, and *mTOR*. These pathways are particularly activated when stimulated by substances such as transforming growth factor (TGF) or endothelin, leading to the growth and remodeling of airway smooth muscle [77].

Recent insights into the aging process have emphasized the critical role of specific signaling pathways and molecular mediators in the tracheobronchial tree. Two major tumor-suppressor pathways—*p53/p21* and *p16INK4a/Rb*—are pivotal in initiating and maintaining cellular senescence. Activation of these pathways occurs in response to DNA damage, telomere shortening, and oxidative stress, leading to irreversible cell cycle arrest and preventing malignant transformation in airway epithelial cells [86,87].

At the same time, chronic exposure to inflammatory stimuli in the aging lung leads to the persistent activation of NF-κB, a key transcription factor that regulates genes involved in inflammation, immunity, and cell survival. NF-κB activation promotes the senescence-associated secretory phenotype (SASP), a pro-inflammatory profile that includes IL-6, IL-8, TNF-α, and MMPs. These molecules not only sustain inflammation but also remodel the extracellular matrix, contributing to airway stiffness and functional decline [88,89,90,91,92,93].

**Figure 2:**
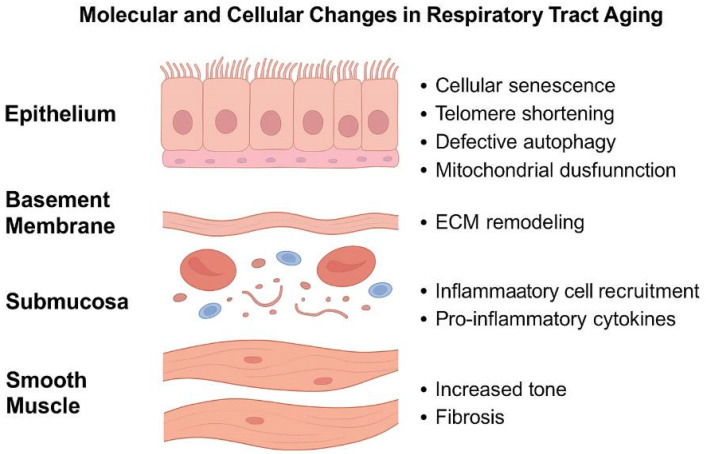
Schematic representation of the key molecular and cellular processes occurring during aging in the respiratory tract. Each histological layer—pseudostratified ciliated epithelium, extracellular matrix (ECM)m submucosa, and smooth muscle—is affected by phenomena such as cellular senescence, telomere shortening, defective autophagy, oxidative stress (ROS), and the senescence-associated secretory phenotype (SASP). These contribute to chronic inflammation, ECM degradation, and tissue remodeling.

Additionally, dysregulation of mTOR (mechanistic Target of Rapamycin) and impaired autophagy are central features of aging in airway smooth muscle and epithelial cells. Normally, autophagy maintains cellular homeostasis by degrading damaged organelles and proteins. In aging tissues, a decline in autophagy, particularly involving *Beclin-1*, *AMPK*, and *ULK1*, leads to mitochondrial dysfunction and accumulation of reactive oxygen species, further promoting senescence and inflammation [41,94,95,96,97,98,99] (Figure 2, Table 2).

Understanding these pathways provides essential context for targeting age-related respiratory decline and offers new perspectives for therapeutic modulation in elderly populations or patients with chronic respiratory diseases such as COPD [9,100] (Table 3).

## 4. Perspectives

After reviewing the literature on the biomolecular changes that occur in the tracheobronchial tree during the aging process, we conclude that there is a close connection between the mechanisms underlying changes in each layer of the airways. These quantitative changes, such as collagen deposition, overproduction of mucins, or the appearance of ARGLS, as well as qualitative changes, such as the alteration of calcium signaling and the decline in mucociliary clearance, are likely part of the functional decline of the respiratory system.

Although numerous studies have investigated the molecular changes that occur with aging, there is a lack of studies that provide a comprehensive understanding of these changes, systematically tracking their appearance over time. Furthermore, there are many respiratory tract diseases where women exhibit a different prognosis and disease progression compared to men or in relation to different hormonal stages [106,107,108]. These hormonal stages occur at various times throughout life, such as before puberty, during puberty, during pregnancy, or menopause. Therefore, investigating hormonal receptors at this level and correlating them with different age stages represents an important direction for future research.

Additionally, with the advent of tools for calculating epigenetic age and exploring how respiratory health could be influenced by the administration of certain anti-aging drugs, such as metformin and rapamycin [109], or physical exercise [110] could provide valuable insights into aging-related changes and potential therapeutic strategies.

A comprehensive overview of the cellular, molecular, and structural changes that occur with aging could provide new therapeutic perspectives, innovative prevention strategies, and potentially even predictive tools for the progression and treatment of acute or chronic conditions affecting the tracheobronchial tree. Understanding these age-related changes in greater detail would allow for more personalized and effective interventions aimed at preserving respiratory function and improving patient outcomes across different age groups.

## 5. Conclusions

Our study highlights the need for comprehensive and unified research across all layers of the bronchial tree walls, both quantitative and qualitative. These aspects have been studied separately until now. Correlating the results of these studies is essential and has the potential to facilitate the development of a complex management protocol for elderly patients. Areas of interest range from gerontology, pulmonology, and cervical-thoracic surgery to intensive care.

## Figures and Tables

**Table 1:** Results of database and register searches, the records identified from databases mentioned in the Methods section, and those excluded according to selection criteria from the Methods section.

Criteria	Stage	Count
1.	Records identified	20
2.	Records removed before screening	6
3.	Records screened	24,765
4.	Records excluded	18,942
5.	Reports sought for retrieval	5823
6.	Reports not retrieved	5
7.	Reports assessed for eligibility	5818
8.	Reports excluded (irrelevant articles)	5390
9.	Reports excluded (not original/review articles)	371
10.	Studies included in review	57

**Table 2:** Key molecules and signaling pathways involved in tracheobronchial aging and their functional roles.

Molecule/Pathway	Affected Layer/Cell Type	Function/Alteration in Aging	Reference(s)
*p53/p21*	Epithelial cells	Senescence induction, cell cycle arrest	[86,87]
*p16INK4a/Rb*	Basal epithelial and ASM cells	Permanent growth arrest, tumor suppression	[87,88]
*NF-κB*	All airway layers	Inflammatory cytokine production (SASP)	[89,90]
mTOR	ASM and epithelial cells	Impaired autophagy, altered cell growth	[91]
*AMPK/Beclin-1*	Epithelial cells	Autophagy regulation, mitochondrial homeostasis	[92,93]
IL-6, IL-8, TNF-α	Immune and epithelial cells	Chronic inflammation, ECM remodeling	[89,94]
*MMP-9*, *MMP-1*	ECM, cartilage	ECM degradation, airway remodeling	[95,96]
*MUC5AC/MUC5B*	Goblet and secretory cells	Mucus overproduction, impaired clearance	[97]
*SOX2*, *KRT5*, *P63*	Basal progenitor cells	Reduced regeneration capacity	[41,98]
ROS	All layers	Oxidative stress, DNA, and mitochondrial damage	[98,99]

**Table 3:** Summary of studies related to tracheobronchial tree aging, with key molecular findings.

Study	Type of Article	Methodology	Model	Key Findings/Molecules
[66]	Original article	Single-cell transcriptomics, proteomics	Mouse	Collagen IV, XIV, XVI alterations in ECM during aging
[38]	Original article	Pharmacological, histological	Human/animal	*KRT5+* basal stem cells; aging effects on epithelial repair niches
[101]	Original article	IHC, gene profiling	Animal	Increased *p63*, club cell markers with aging
[102]	Original article	Proteomics, pathway analysis	Human	*MMP-8*, *-9*, *-10* upregulated with age; ECM remodeling
[103]	Review	Literature synthesis	-	Pro-inflammatory cytokines (IL-6, IL-1, TNF-α); secretory senescence
[56]	Original article	Mucin quantification, ciliary beat	Animal	Reduced *MUC5B* in old mice; decreased mucociliary clearance
[104]	Original article	Transcriptomics, proteomics	Human	COL6A1, LUM, FBLN2; structural ECM differences in aging lungs
[41]	Original article	IHC, IF	Human	*SOX2+*, *KRT5+*, *P63+* stem cell senescence in aging lung
[105]	Review	Literature review	-	Aging effects on lung regeneration and epithelial stem cells
[16]	Review	Literature review	-	Molecular basis of lung aging and disease susceptibility

## Data Availability

Data are contained within the article.
